# Improving Cardiovascular Outcomes: The Era of Personalized Therapy in Atherosclerosis

**DOI:** 10.3390/jcm11113077

**Published:** 2022-05-30

**Authors:** Anna Kabłak-Ziembicka

**Affiliations:** 1 Department of Interventional Cardiology, Institute of Cardiology, Jagiellonian University Medical College, Św. Anny 12, 31-008 Kraków, Poland; kablakziembicka@op.pl; 2 Noninvasive Cardiovascular Laboratory, The John Paul II Hospital, Prądnicka 80, 31-202 Kraków, Poland

Data from the European Society of Cardiology report that cardiovascular disease (CVD) is responsible for app. 40% of the mortality in Europe, with atherosclerosis being a major contributor to case fatality [1]. Progression of atherosclerotic lesions is a time-dependent condition leading to life-shortening clinical events, such as myocardial infarction, ischemic stroke, claudication and renal failure, or intestinal ischemia [2,3,4].

The maxima of Niccolò Machiavelli published in “The Prince”, in the year 1513, that ‘… at the beginning a disease is easy to cure but difficult to diagnose; but as time passes, not having been recognized or treated at the outset, it becomes easy to diagnose but difficult to cure.’; this is particularly true with regard to atherosclerosis [5].

In accordance with this maxima, the first step for the evaluation of cardiovascular risk in the individual person is a development of the Framingham score and European SCORE, introduced in the years 1998 and 2003, respectively [6,7].

Prevention strategies in cardiovascular disease, particularly in atherosclerosis, are associated with over 50% cardiovascular risk reduction, compared with 5–10% risk reduction obtained with the surgical and endovascular interventions [8,9]. Capewell et al. observed 341,745 fewer cardiac deaths in the year 2000, and a 61% risk reduction in those patients who obtained a traditional cardiovascular risk factor-reduction strategy, compared with the year 1980 when prevention strategies were hardly available [8].

Since then, cardiovascular prevention scores based on atherosclerosis risk factors are continuously improved and developed [10]. Novel approaches include incorporation of inflammatory and imaging parameters into traditional scores, such as subclinical atherosclerosis measures, endothelial dysfunction, air pollution, fibrin clot properties, inflammation, cytokines, and other biomarkers [10,11,12].

The identification of new risk factors, and their associations with atherosclerosis progression or triggering adverse cardiovascular events, led to the introduction of novel, more effective treatment agents [13,14]. Introduction of hypercholesterolemia management with statins, and more recently with protein convertase subtilisin/kexin type 9 (PCSK9) inhibitors, and/or diabetes treatments with new noninsulin glucose-lowering drugs, and many others are effective in a reduction of cardiovascular morbidity and mortality [13,14]. In consequence, improved management strategies and decision-making algorithms are continuously introduced.

Interventional treatment, although less effective in adverse cardiovascular event rate reduction compared with prevention strategies, also have a role in advanced atherosclerosis management [8].

As previously evidenced, combining risk factors management focused on achieving therapeutic targets with an intervention on athero-occlusive disease enhances long-term procedure results [15].

Accomplishing both treatment goals through achieving optimal cardiovascular risk factors control and restoring arterial blood flow to the supplied organs like the brain, the kidneys, and the heart may amplify the impact of both strategies on cardiovascular outcomes in the individual patient [15,16].

The important clinical issue is the management of patients with so called polyvascular disease, defined as the presence of atherosclerotic lesions within two or more arterial beds [17,18,19]. This subgroup of patients requires special attention with regard to multi-factorial medical therapies, usually combined with staged interventional therapies aimed at mitigating ischemic events in this high-risk population [17,18,19].

Thus, in the part II of this Special Issue on ‘The prevention and Treatment of Atherosclerosis’, review and research papers on the novel prevention measures and the diagnostic and therapeutic achievements in optimizing atherosclerosis management are welcome. Of utmost importance is contemporary research on microparticles, non-coding RNAs, proteomic characterization, chemokines, and fibrin clotting that offer molecular characteristics of athero-thrombosis.

In Part II of Special Issue, we also encourage the submission of papers addressing the favorable and adverse outcomes of the interventional and pharmacological treatment strategies in patients with coronary, extra coronary, and poly vascular steno-occlusive disease. Both safety and the procedure optimization guarantee favorable outcomes. There is still a field for new stent and equipment technologies, new surgical and endovascular techniques, supervision of endovascular procedures with IVUS, OCT, functional flow assessment, or cell therapy [20].

Of great interest, there are research papers on achieving goals in cardiovascular risk factors prevention and treatment, cardiovascular risk stratification and algorithms, new treatments of diabetes and hyperlipidemia for cardiovascular outcomes, and imaging tools to control atherosclerosis reduction and progression [21]. 

I believe that we are entering the era of personalized therapies. There is time for individually tailored treatment for occlusive atherosclerotic lesions in coronary and extra coronary territories, synchronizing interventional and prevention strategies in athero-occlusive disease [22].
